# The Biological Clock: A Pivotal Hub in Non-alcoholic Fatty Liver Disease Pathogenesis

**DOI:** 10.3389/fphys.2018.00193

**Published:** 2018-03-15

**Authors:** Gianluigi Mazzoccoli, Salvatore De Cosmo, Tommaso Mazza

**Affiliations:** ^1^Division of Internal Medicine and Chronobiology Unit, Department of Medical Sciences, IRCCS “Casa Sollievo della Sofferenza”, San Giovanni Rotondo, Italy; ^2^Bioinformatics Unit, IRCCS “Casa Sollievo della Sofferenza”, San Giovanni Rotondo, Italy

**Keywords:** NAFLD, clock gene, circadian, rhythm, metabolism, chronodisruption

## Abstract

Non-alcoholic fatty liver disease (NAFLD) is the most frequent hepatic pathology in the Western world and may evolve into steatohepatitis (NASH), increasing the risk of cirrhosis, portal hypertension and hepatocellular carcinoma. NAFLD derives from the accumulation of hepatic fat due to discrepant free fatty acid metabolism. Other factors contributing to this are deranged nutrients and bile acids fluxes as well as alterations in nuclear receptors, hormones, and intermediary metabolites, which impact on signaling pathways involved in metabolism and inflammation. Autophagy and host gut-microbiota interplay are also relevant to NAFLD pathogenesis. Notably, liver metabolic pathways and bile acid synthesis as well as autophagic and immune/inflammatory processes all show circadian patterns driven by the biological clock. Gut microbiota impacts on the biological clock, at the same time as the appropriate timing of metabolic fluxes, hormone secretion, bile acid turnover, autophagy and inflammation with behavioural cycles of fasting/feeding and sleeping/waking is required to circumvent hepatosteatosis, indicating significant interactions of the gut and circadian processes in NAFLD pathophysiology. Several time-related factors and processes interplay in NAFLD development, with the biological clock proposed to act as a network level hub. Deranged physiological rhythms (chronodisruption) may also play a role in liver steatosis pathogenesis. The current article reviews how the circadian clock circuitry intimately interacts with several mechanisms involved in the onset of hepatosteatosis and its progression to NASH, thereby contributing to the global NAFLD epidemic.

## Introduction

Non-alcoholic fatty liver disease (NAFLD) originates from disproportionate triglyceride deposition in hepatocytes, which contributes to more than 5–10% percent of the liver's weight, concurrent to major macrovesicular steatosis, all in the absence of excessive alcohol consumption (Kanwal et al., 2016). Liver steatosis is the hepatic manifestation of metabolic syndrome and represents the primary cause of chronic liver disease in the Western world (Bonora and Targher, 2012; Wong et al., 2015; Kanwal et al., 2016). NAFLD affects at least 30% of the general population, being present in more than 60% of people with obesity and embracing different histological patterns, which range from uncomplicated steatosis to steatohepatitis (NASH) (Bonora and Targher, 2012; Wong et al., 2015; Kanwal et al., 2016). NASH is regarded as the most common likely indication for liver transplantation over the next decade worldwide, and is a prevailing end-stage kidney disease and cardiovascular disease risk factor (Bonora and Targher, 2012; Musso et al., 2014; Wong et al., 2015; Wu et al., 2016). A major, albeit not indispensable, role in NAFLD pathogenesis is played by the reduced secretion of insulin, coupled to insulin insensitivity. Insulin crucially regulates blood glucose levels upholding glycogen synthesis in the fed state and restraining hepatic glucose production during fasting. Insulin resistance is intimately linked to hyperinsulinemia, which boosts hepatic lipogenesis and leads to hepatosteatosis. In turn, the buildup of different lipid species in the liver elicits inflammation or endoplasmic reticulum (ER) stress, turning on numerous protein kinase cascades, which in due course produce insulin signaling disturbances further enhancing insulin resistance (Titchenell et al., 2017).

Augmented hepatic triglycerides levels may be caused by undue free fatty acid (FFA) delivery to the liver, especially after eating, which is related to adipose tissue insulin resistance in people with obesity (Lambert et al., 2014; Titchenell et al., 2017). In addition, increased *de novo* lipogenesis, driven by hyperinsulinemia and unwarranted carbohydrate intake causing triglycerides overload, may not be balanced by adequate increases in very low density lipoprotein (VLDL) secretion and changes in levels of β-oxidation (Lambert et al., 2014; Titchenell et al., 2017). In recent decades dietary habits have progressively changed, leading to the consumption of low-nutrient, energy-dense foods and beverages containing high levels of sugars and fats. This has also been coupled to decreased physical activity. These factors have triggered the increased prevalence of metabolic diseases, driven by energy imbalance.

Energy homeostasis is maintained by signaling interactions among metabolically sensitive tissues, such as the intestine, pancreas, and adipose tissue. However, the liver is the master hub that regulates conflicting metabolic processes, such as glycolysis/gluconeogenesis and lipogenesis/fatty acid β-oxidation (Corkey and Shirihai, 2012; Paschos et al., 2012). Multiple organs may be implicated in dysmetabolism onset and progression and the molecular mechanisms may entail signal transduction pathways in pancreatic islets, liver, adipose tissue, as well as brain, gut, vasculature, and muscle (Corkey and Shirihai, 2012; Paschos et al., 2012). Metabolic derangements can be initiated in any of these organs and tissues, with blood-borne molecules having metabolic effects in other tissues and organs, in an ongoing process of reciprocated interactions (Corkey and Shirihai, 2012; Paschos et al., 2012). This coordination among specialized tissues to manage essential and dedicated cellular processes under homeostatic as well as stress conditions is maintained through long-distance inter-organ communication networks hardwired by humoral factors, such as hormones (i.e., insulin, adipocytokines, glucocorticoids), peptides, proteins, and the sympathetic and parasympathetic fibers of the autonomic nervous system (ANS; Corkey and Shirihai, 2012; Paschos et al., 2012). A crucial role in the molecular regulation of metabolic tissues is played by metabolites (e.g., glucose, fatty acids, sterols, and bile acids), which are sensed by nuclear receptors and regulate nutrient directed signal transduction pathways (Mazzoccoli et al., 2014). Intestinal nutrient digestion and extraction is a primary process in body homeostasis, which prompts intermediary metabolism. In addition, the intestine secretes enteroendocrine hormones and harbors the gut microbial community, with the latter being increasingly recognized to have considerable importance in the regulation of metabolic processes (Sanz et al., 2015). The temporal dimension is a crucial player in NAFLD pathogenesis considering that the processes involved in hepatosteatosis onset and progression show time-related fluctuations. Altered resonance between geophysical cycles and the array of physiological rhythms bringing about internal timing is gradually more acknowledged as involved in the global NAFLD epidemic.

## The biological clock plays a crucial role in the development of hepatic steatosis

Liver metabolic pathways and bile acid synthesis as well as basic cell functions, such as autophagy, are regulated by circadian processes, which are, in turn, modulated by the gut microbiome. Circadian regulation of patterns of physiological functions as well as hormone secretion, metabolic fluxes, bile acid turnover, autophagic and inflammatory processes, coupled to the behavioral timing of sleep/wake and fasting/feeding cycles are crucial to preserve liver health and circumvent metabolic derangement (Mazzoccoli et al., 2012; Cermakian et al., 2013; Rosselot et al., 2016). Energy regulation is crucial to survival and requires the monitoring of blood-borne nutrient levels, the triggering of food seeking and stimulated insulin secretion to prompt satiety. Inappropriate nutritional signaling triggers suboptimal eating behavior, exemplified by alterations in leptin signaling in the C57BL/KsJ-*db/db* mouse (Bogdanov et al., 2014), and flawed signaling related to systemic responses to misread cues could set off metabolic derangement (Corkey and Shirihai, 2012).

### The circadian timing system

The 24-h rhythms hallmarking feeding behavior, locomotor activity, and biophysical functions are managed by the circadian timing system, a hierarchical multilevel supra-physiological network encompassing biological oscillators in the suprachiasmatic nuclei (SCN) of the hypothalamus, driving cell-autonomous, and self-sustained biological oscillators in peripheral tissues (Moore, 2013). This circadian time-keeping system guarantees organism adaptation to predictable circadian changes in light-dark, perceived by melanopsin-containing intrinsically photosensitive retinal ganglion cells responsive to blue light in the range of 460–480 nm and conveyed through glutamatergic fibers of the retino-hypothalamic tract (Moore, 2013). The SCN entrain peripheral oscillators *via* output signals that may be humoral (cortisol, melatonin), physical (temperature changes), and neural (ANS fibers) to harmonize food intake behavior, gastrointestinal system activity, and transcription of genes enriching pathways involved in glucose and lipid metabolism (Bosler et al., 2015). In mammals, the biological clocks coordinate oscillations in nutritional status and redox balance with rhythmic variations of the transcriptome and metabolome, with metabolomic studies showing circadian fluctuation of a wide array of hepatic metabolites (Dallmann et al., 2012; Eckel-Mahan et al., 2012).

The interplay among the circadian timing system and inter-organ communication networks drives the regulation of integrated physiology though molecular mechanisms and input/output signaling pathways allowing resonance between the temporal coordination of metabolism and geophysical time. In this regard, time-dependent profiling of mouse transcriptomes shows how organ-to-organ coexpression networks and circadian rhythms bring about a complex periodic orchestration of gene pathways throughout the body. Such processes may become desynchronized in disease, including inter-organ processes, suggesting efficacy from chronotherapeutic strategies (Zhang et al., 2014). Circadian misalignment is not uncommon due to a variety of conditions, including exposure to artificial light-at-night, night-shift work, sleep restriction, sleep fragmentation, and frequent time-zone travels, which may all contribute to metabolic dysregulation and consistently propel the worldwide spread of metabolic diseases (Pan et al., 2011; Buxton et al., 2012). Interestingly, over the past 20 years, obesity prevalence correlates with increased exposure to artificial light-at-night, implicating this environmental factor to the global obesity pandemic Besides, long-established strategies, such as low-calorie diet combined with increased physical activity, are helpful in reducing hepatosteatosis severity at the individual level (Fock and Khoo, 2013), but lifestyle behaviors modification and dietetic changes have been ineffective in restraining the metabolic disease epidemics at general population level in the past two decades, suggesting the involvement of underrated mechanisms in NAFLD pathogenesis, such as exposure to artificial light during the night-time hours and social jet lag (misalignment of biological and social time). Specific genetic changes and mutations of circadian genes could give rise to augmented lipid synthesis and uptake, and/or reduced free fatty acid β-oxidation and triglyceride export, but they are uncommon and would not account for the greater part of NAFLD cases. The multifaceted interaction among the metabolic pathways involved in bile acids, glucose, and lipids turnover as well as cellular processes comprising autophagy, inflammation and their regulation by the biological clock in response to nutrients, bile acids, hormones, nuclear receptors, or gut microbiota is central in the regulation of metabolism and in the manifestation of hepatic steatosis (Table 1).

**Table 1:** NAFLD pathogenesis and the biological clock: a crucial interplay.

Lifestyle behaviors, including overconsumption of energy-dense foods and diminished physical activity, increase the prevalence of NAFLD in affluent and non-affluent countries, which correlates with the rise of obesity, metabolic syndrome, and type 2 diabetes mellitus.
Energy balance in the body is coordinated by the internal timing system, with metabolic information conveyed through nutrient flux, humoral factors, and autonomic nerve fibers. The liver integrates and processes signals derived from other metabolically active organs.
Exposure to artificial light-at-night or jet lag causes chronodisruption and deranged time-related energy homeostasis, which may play a role in the pathogenesis of hepatosteatosis as well as the global NAFLD epidemic.
Altered time-related oscillations of nutrients and bile acids, *via* metabolic and inflammatory signaling pathways receptors, are crucial to the pathophysiological underpinnings of NAFLD and its progression to NASH.
Dysregulated interactions between the circadian timing system and interorgan communication networks disrupt the chronological harmonization of behavioral and physiological processes. Circadian disruption provokes desynchronization of the metabolic and inflammatory signaling pathways, with respect to geophysical and environmental cues.
Autophagic and inflammatory processes, nutrient flux, bile acids turnover, hormone secretion, nuclear receptors, and gut microbiota are driven by the molecular clockwork. The complex interplay among dietary signals, bile acids, immune-metabolic pathways, autophagy, gut microbiota, and the biological clock are important treatment and prevention targets.

### The cogwheels of the molecular clockwork

The mammalian biological oscillators are operated by molecular clocks maneuvred by a transcriptional-translational feedback loop (TTFL) that is regulated by circadian genes and proteins and oscillates with a 24-h rhythmicity (Takahashi, 2017). The TTFL positive limb is modulated by the bHLH (basic Helix-Loop-Helix) and PAS [Drosophila Period (Per), human Arnt, and Drosophila Single-minded (Sim)] containing transcription factors Clock (circadian locomotor output cycles kaput), or its paralog Npas2 (neuronal PAS domain protein 2), and Arntl/Bmal1 (aryl-hydrocarbon receptor nuclear translocator-like/brain and muscle aryl-hydrocarbon receptor nuclear translocator-like 1) or its homolog Arntl2/Bmal2, which heterodimerize and bind hexameric DNA sequences, referred to as E-Boxes, on Period (Per1-3) and Cryptochrome (Cry 1-2) genes (Takahashi, 2017). Per and Cry proteins manage the negative limb of the TTFL, dimerizing and forming a repressor complex that enters the nucleus and inhibits Clock or Npas2/Arntl 1-2 transcriptional activity. The expression of the positive limb transcription factors Arntl and Clock is driven by the nuclear receptors RORα/β/γ and Rev-erbα/β, which compete at specific response elements (RORE) in the promotors of these genes. In turn, the transcription of circadian genes involves binding of the Arntl/Clock heterodimer to target gene promoter elements, release/recruitment of coactivators or corepressors, epigenetic modifications (acetylation/deacetylation, methylation/demethylation cycles), chromatin remodeling and in due course interaction with the polymerase II complex (Takahashi, 2017).

In insects and flies a component of the molecular clockwork is embodied by Timeless (Tim), which is conserved in mammals and impacts embryonic development, cell cycle progression, DNA replication and DNA damage response (Mazzoccoli et al., 2016). The functioning of the molecular clockwork depends on proper turnover of circadian proteins and their post-translational modifications, including acetylation, sumoylation, glycosylation, and especially phosphorylation (Robles et al., 2017). Per and Cry proteins are substrates for at least three different enzymes: casein kinase (CK)Iδ, CKIε, or 5′-adenosine monophosphate-activated protein kinase (AMPK) and glycogen synthase kinase (GSK)-3β. In particular, CKIδ and CKIε target Arntl as well as Per/Cry proteins controlling their nuclear translocation and tagging them for proteasomal degradation, AMPK targets Cry proteins and GSK-3β targets Arntl, Clock, Cry, Per, and Rev-erb proteins (Robles et al., 2017). Notably, intracellular glucose levels impact on the molecular clockwork through the hexosamine/O-GlcNAc pathway: Arntl and Clock are rhythmically O-GlcNAcylated with reduced ubiquitination, protein stabilization, and increased expression of target genes (Li et al., 2013).

In addition to these processes, the NAD+-dependent histone and protein deacetylase, SIRT1, counters the acetyltransferase activity of Clock, binds Arntl/Clock heterodimer in a circadian manner, and promotes the deacetylation and degradation of Per2, allowing high-magnitude circadian transcription of several core clock genes, including Arntl, Per2, and Cry1 (Takahashi, 2017). SIRT1 and another NAD+-sensitive enzyme, poly (ADP-ribose) polymerase 1 (PARP-1), play key roles in the coordination of peripheral clocks and metabolic cycle phases (Asher et al., 2010). AMPK boosts SIRT1 activity through cellular NAD+ levels augmentation, with the enhanced deacetylation and activity modulation of peroxisome proliferator-activated receptor-γ coactivator 1α and the forkhead box O1 (FOXO1) and FOXO3a transcription factors. Such processes highlight the cooperative biological effects of AMPK and SIRT1 on energy metabolism (Cantó et al., 2009).

**Figure 1:**
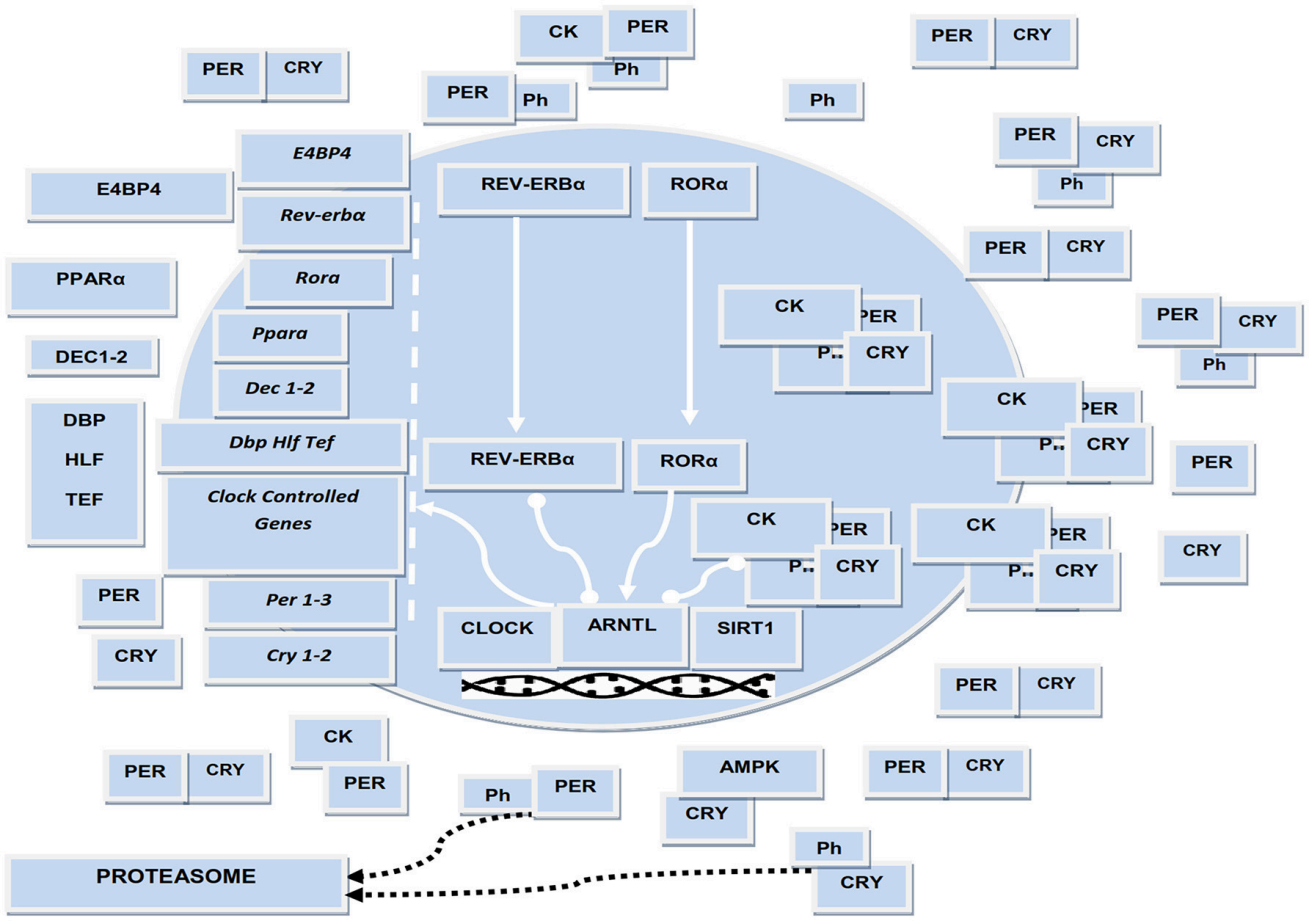
Schematic representation of the biological clock. The several cogs of the molecular clockwork are connected by lines representing interaction, arrow-headed lines representing activation, bar-headed lines representing inhibition. ARNTL, CLOCK, and RORA encode transcriptional activators; PER1/2, CRY1/2, and Rev-erbα encode transcriptional repressors; CK and Ph symbolize casein kinases and inorganic phosphate operating by post-translational modification of circadian proteins. ARNTL drives the expression of downstream genes encoding transcriptional activators, such as DEC1-2, DBP, TEF, HLF, PPARA. CLOCK acetyltransferase activity and SIRT1 deacetylase activity is crucial for the functioning of the biological clock. Molecular interactions were manually retrieved and curated from the scientific literature.

Arntl drives the circadian expression of the PARbZip transcriptional activators DBP, HLF, and TEF, while the interplay of Rev-erbs with RORs on RORE drives the cyclic expression of the transcriptional repressor E4BP4 (or NFIL3). These factors play a key role in controlling the expression of a huge number of clock controlled genes as well as in regulating drug metabolism and xenobiotic responses (Mazzoccoli et al., 2012). Arntl expression is also regulated by a supplementary feedback loop operated by the bHLH proteins Dec1 (or BHLHE40) and Dec2 (or BHLHE41), whose expression is driven by Arntl/Clock transcriptional activator complex binding to CACGTG E-boxes in their promoters and, subsequently, restrained by competitive DNA binding with Arntl/Clock heterodimer (Kato et al., 2014). Dec 1 and Dec2 modify the interaction of retinoid X receptor (RXR)α with cofactor proteins as well as RXR nuclear receptors heterodimerization and ligand-dependent transactivation, impacting transcriptional networks entailing other nuclear receptors driving genes that enrich lipid and glucose metabolic pathways and interplaying with the molecular clockwork (De Cosmo and Mazzoccoli, 2017; Figure 1).

## The biological clock controls intermediary metabolism and autophagy

Glucose levels in the peripheral blood are maintained in tight physiological range, but show fluctuations with 24-h periodicity related to feeding patterns as well as to oscillations in glucoregulatory hormones (Mazzoccoli et al., 2012). Glucagon is secreted by α pancreatic cells and counters the effects induced by insulin. Glucogon shows circadian changes in the rat plasma (Ruiter et al., 2003) and during fasting turns on the CREB/CRTC2 transcriptional complex, which binds to the *Arntl* promoter and promotes its expression (Sun et al., 2015). Glucagon-like peptide-1 (GLP-1) is an incretin hormone secreted by the intestinal L cells, and operates as a link between the gut and pancreatic β-cells. Notably, GLP-1 is produced with a circadian rhythmicity (Gil-Lozano et al., 2014). Healthy humans show circadian rhythms of insulin sensitivity and insulin secretion, whilst the mouse and human Langherans islet β-cells harbor autonomous and self-sustained circadian oscillators that drive insulin synthesis and release (Sadacca et al., 2011; Eckel-Mahan and Sassone-Corsi, 2013; Pulimeno et al., 2013; Perelis et al., 2015). Experiments performed in mouse models as well as in human islets isolated from the pancreas of brain-dead multi-organ donors show insulin release is rhythmically regulated by peripheral pancreatic β-cell clocks, even in isolated perfused condition (Saini et al., 2016). Accordingly, whole body *Arntl*^−/−^ mice show glucose intolerance, hypoinsulinemia, an increased respiratory quotient, reduced fat storage, increased circulating fatty acids, increased ectopic fat formation in liver and muscle, and hypoinsulinemia, whilst mouse liver specific *Arntl* knock-out induces hypoglycemia in the resting state (Lamia et al., 2008; Marcheva et al., 2010; Shimba et al., 2011). Furthermore, *Arntl* was found necessary for β-cell adaptative growth, survival, and metabolic adjustment to diet-induced obesity in mice (Rakshit et al., 2016), whilst whole-body *Clock* mutant mice (*Clock*^Δ19^) are characterized by age-dependent damped feeding rhythms, obesity, hyperphagy, hyperlipidemia, hyperglycemia, hypoinsulinemia, and hepatic steatosis (Rudic et al., 2004; Turek et al., 2005; Marcheva et al., 2010). Notably, the *Clock*^Δ19^ mutant mouse was bred on a C57BL/6J strain background lacking melatonin production, with melatonin limiting basal and glucose-stimulated insulin secretion.

Moreover, double *Cry1* and *Cry2* knockout mice show glucose intolerance and chronically elevated levels of circulating corticosterone with augmented glucocorticoid-dependent transactivation in the liver (Lamia et al., 2011), which is coupled to hyperglycemia induced by defective restraint of glucagon-stimulated hepatic gluconeogenesis (Zhang et al., 2010). Accordingly, genome-wide association studies performed in humans found elevated fasting glucose levels and altered response of energy expenditure to weight-loss diets (measured as respiratory quotient and resting metabolic rate) in carriers of *CRY2* rs11605924 variant as well as rs10830963G allele of *MTNR1B* gene, encoding the MT2 melatonin receptor (Mirzaei et al., 2014; Lane et al., 2016; Tuomi et al., 2016). Carriers of *MTNR1B* risk variant show amplified MT2 receptor expression in pancreatic β-cells, as well as delayed dim light melatonin offset and a more prolonged melatonin secretion. Such alterations increase the risk of food intake coincident with amplified morning melatonin levels, coupled to decreased glucose tolerance (Lane et al., 2016; Tuomi et al., 2016).

Rev-erbs and RORs, working as transcriptional repressors and activators, respectively, control the circadian expression of numerous target genes encoding proteins involved in lipid metabolism. Accordingly, *Rev-erb*α^−/−^ mice are hallmarked by elevated VLDL triglyceride levels linked to increased ApoC-III expression in the liver. In contrast, the RORα mutant mouse (*ROR*α^*sg*/*sg*^, Staggerer mouse), in spite of greater food intake, has decreased body fat, smaller fat cells in brown and white adipose tissue, lower liver triglyceride levels and is not prone to hepatic steatosis (Solt et al., 2011). Whole-body double knockout *Rev-erb*α and *Rev-erb*β mice show a phenotype characterized by altered rhythmic expression of the core clock and lipid homeostatic gene networks leading to altered circadian wheel-running behavior, hyperglycemia, hyperlipidemia and hepatic steatosis (Bugge et al., 2012; Cho et al., 2012). The nuclear receptor corepressors (N-CoR)/Histone Deacetylase 3 (HDAC3) complex is necessary for repression by Rev-erbα, with liver-specific HDAC3 knockout causing deregulated lipid metabolism and hepatic steatosis in the mouse (Feng et al., 2011).

**Figure 2:**
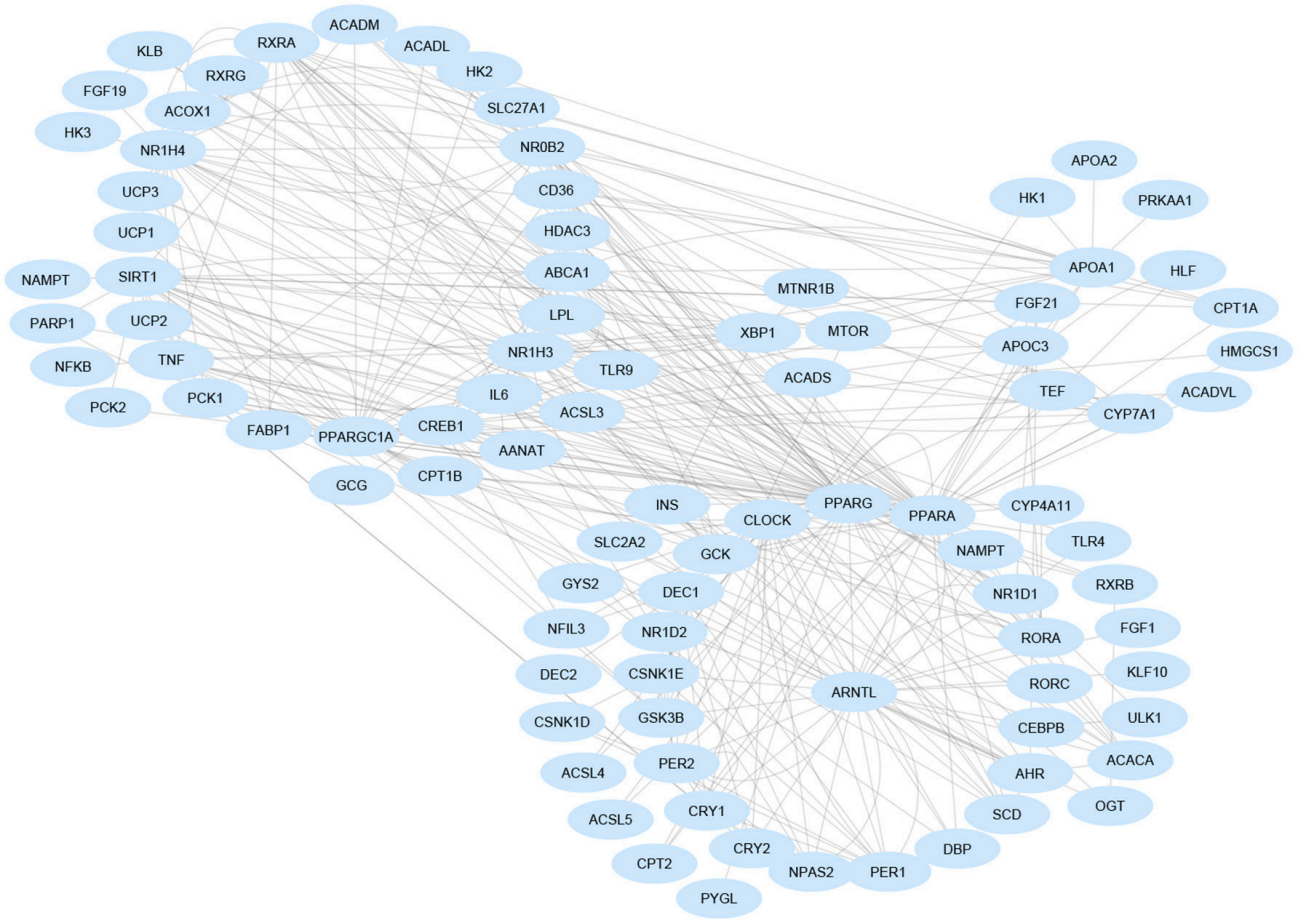
Gene regulatory network involved in non-alcoholic fatty liver disease pathogenesis. Gene are nodes of the network and are visualized as blue-colored ellipses. Most nodes are involved in multiple signaling pathways and are clustered in functionally related groups. Interaction data are retrieved from the Ingenuity Knowledge Base (QIAGEN Inc., https://www.qiagenbioinformatics.com/). Graphical layout was obtained by Cytoscape 3.4.0 (www.cytoscape.org/).

Rev-erbα also impacts on bile acid metabolism through the liver X receptor (LXR or NR1H3), whose transcriptional activity changes upon binding of rhythmically synthesized oxysterols and which temporally interacts with the farnesoid X receptor (FXR or NR1H4), the nuclear receptor capable of binding bile acids and showing circadian rhythmicity in the liver. LXRs and FXRs drive, positively and negatively, respectively, the 24-h periodicity of CYP7A1, the rate-limiting enzyme in bile acid synthesis. The cyclic expression of *Cyp7a1* is driven also by Rev-erbα, DBP/E4BP4, and DEC2, contributing to the regulation of amplitude, phase and wave form over the 24-h profile, controlling gene expression by the use of Rev-RORE, DBP/E4BP4-binding elements, and E-boxes, respectively. Liver expression of E4BP4 and the orphan nuclear receptor small heterodimer partner (SHP), both negative regulators of *Cyp7a1* expression, are increased in *Rev-erb*α knock-out mice with a minor impact on the amassing of hepatic fat. This is due to compensation by Rev-erbβ (Duez et al., 2008), whereas total hepatic Rev-erb deficiency significantly increases hepatosteatosis (Bugge et al., 2012). In turn, SHP restrains *Npas2* gene transcription and promoter activity, and inhibits Rorγ transactivation and Rorα action, the latter driven by its interaction with Rev-erbα. Npas2 binds rhythmically to the *Shp* promoter, which drives the circadian expression of *Shp* with elevating NAD+ levels. Accordingly, Npas2 deficiency causes severe hepatosteatosis in *Shp(–/–)* mice through derangement of lipoprotein metabolism (Lee et al., 2015; Figure 2).

Autophagy plays a crucial role in NAFLD pathogenesis, with factors modulating autophagy proposed to have therapeutic utility (Lin et al., 2013; Fucho et al., 2014; González-Rodríguez et al., 2014; Jung et al., 2015; Martinez-Lopez and Singh, 2015; Wang et al., 2015). Autophagy is a complex, highly conserved, ATP-dependent cellular process taking place in all eukaryotic cells in response to different stressors, including fasting, changes in cell volume, oxidative stress, damaged protein accumulation, hormonal signals, and xenobiotic exposure (Kaur and Debnath, 2015). Autophagy contributes to the regulation of many biochemical processes, including: remodeling during development and cell differentiation; intracellular protein degradation; amino acid production during stress and nutrient shortage; the replacement of cytomembranes and organelles, such as mitochondria and peroxisomes; and the control of programmed, non-apoptotic cell death (Kaur and Debnath, 2015). Murine studies show autophagic vacuole numbers and autophagy activity, as well as autophagy gene expressions, to show circadian oscillation in the liver, which is driven by the basic leucine zipper transcription factor C/EBPβ and is under circadian regulation by the molecular clockwork (Ma et al., 2011). Timing cues driving transcriptional regulation of autophagy circadian patterns involve both the biological clock and nutritional signals (Ma et al., 2011).

Circadian rhythmicity of autophagy flux and autophagy gene expression is dampened in liver-specific *Arntl* null mice, implying a clock-related cell-autonomous mechanism. Nutritional cues entrain the biological oscillators of peripheral tissues. This is exemplified by time restricted feeding (Table 2), which resets the phase of peripheral clocks in rodents without affecting the SCN. This suggests a phase shift in the autophagy rhythm that is dependent upon feeding-related biological clock resetting (Atger et al., 2015). Rhythmic autophagy activation could maintain nutrient and energy homeostasis, reshape proteomes, and organelles, and attain temporal compartmentalization of tissue metabolism. Given that the expression of genes involved in glucose metabolism, *de novo* lipogenesis, cholesterol biosynthesis, and fatty acid β-oxidation oscillate in the liver, coordination of circadian patterns of metabolic cycles with autophagy rhythms could optimize nutrient supply for storage or utilization (Ma et al., 2012).

**Table 2:** Dietary patterns impacting the biological clock.

**Caloric restriction**, a dietary regimen with 20–40% daily calorie intake decrease, whilst maintaining meal frequency to avoid malnutrition or essential nutrients deficiency.
**High-fat diet**, a diet regimen containing a higher percentage of fat (≥45% energy by fat) with respect to a normal diet (≤10% fat).
**Fasting mimicking diet**, a plant-based diet program planned to accomplish fasting-like effects while supplying micronutrient nourishment (vitamins, minerals, etc.), whilst reducing the challenge of fasting. It consists of proprietary vegetable-based soups, energy bars, energy drinks, chip snacks, chamomile flower tea, and a vegetable supplement formula tablet; the diet program consists of a 5 day regimen: day 1 of the diet supplies 1.090 kcal (10% protein, 56% fat, 34% carbohydrate), days 2–5 are identical in formulation and provide 725 kcal (9% protein, 44% fat, 47% carbohydrate).
**Intermittent fasting**, consumption of water exclusively or very low calorie (~500 kcal/die) diet for less than 24 h followed by normal feeding for 1–2 days.
**Periodic fasting**, a dietary pattern entailing 2 or more days of no food consumption repeated after no <1 week of regular feeding.
**Time restricted feeding**, a daily eating pattern in which all nutrient intake occurs within a few hours (usually <12 h) every day, with no overt attempt to alter quantities, proportions, variety, or combination of different foods, drinks, and nutrients.
**Ketogenic diet**, a high-fat diet with sufficient protein and very-low-carbohydrate intake triggering a switch to fatty acid β-oxidation as an energy source.

During glucose starvation, AMPK promotes autophagy by activating Ulk1, in a process involving phosphorylation of Ser 317 and Ser 777 residues, whilst this is restrained when nutrients are abundant *via* mammalian target of rapamycin (mTOR) (Kim et al., 2011). AMPK directly impacts the functioning of the biological clock, with Arntl negatively modulating mTOR oscillating activity (Khapre et al., 2014). Autophagy is crucial for cellular metabolic homeostasis, with the transcriptional control of autophagy being managed by the nutrient-sensing nuclear receptors peroxisome proliferator-activated receptor-α (PPAR-α) and FXR, which are driven by the biological clock and hepatically induced under nutrient-deprived conditions as well as in the fed state, respectively (Lee et al., 2014; Seok et al., 2014). The FXR transcriptionally represses autophagy in the liver upon feeding, opposing cAMP response-element binding protein (CREB)-mediated activation of autophagy during fasting. Indeed, autophagic genes are hallmarked by FXR binding site enrichment at transcription start sites and CREB binding site enrichment upstream (Lee et al., 2014; Seok et al., 2014).

On the whole, these data pinpoint to the pivotal role played by the molecular clockwork in hepatic steatosis pathogenesis, considering that the processes involved in intermediary metabolism and autophagy are hallmarked by a circadian pattern of variation and their rhytmicity is impacted by feeding–fasting cycles reverberating physiological and behavioral rhythmicity driven by the biological clock.

## From NAFLD to NASH: the role of the biological clock

NAFLD may progress to NASH, hallmarked by steatosis, necroinflammation, and fibrosis, with varying degrees of severity. This can then progress to cirrhosis, portal hypertension and hepatocellular carcinoma (HCC). NASH may also arise independently of simple hepatic steatosis, driven by a diverse array of pathogenic events (Yilmaz, 2012). The progression of NAFLD to NASH, fibrosis and HCC is a pattern more commonly evident in obese humans and which is paralleled in wild-type mice challenged with chronic jet lag (Kettner et al., 2016). Hepatic circadian homeostasis regulation prevents this progression to HCC, including from the dysregulation of nuclear receptors involved in cholesterol/bile acid turnover and xenobiotic metabolism (Kettner et al., 2016). All the mechanisms involved in NAFLD pathogenesis and its evolution to NASH are handled by the molecular clockwork and include endoplasmic reticulum (ER) stress, lipotoxicity, insulin resistance, mitochondrial dysfunction, oxidative stress, adipose tissue dysfunction, deranged control of innate immunity, and cytokine secretion as well as alterations in the gut–liver axis.

### Endoplasmic reticulum (ER) stress and NAFLD

The biological clock is an important regulator of hepatic metabolism as well as driving ultradian rhythms of the IRE1α (ERN1)-XBP1 pathway important to the unfolded protein response (UPR). The UPR is a biological process activated during ER stress and is stimulated by incorrectly folded protein accumulation (Cretenet et al., 2010). The UPR reinstates ER homeostasis and operates by arresting protein translation, degrading improperly folded proteins, and switching on signaling pathways to augment protein folding chaperones. ER stress plays a crucial role in metabolic homeostasis, with its derangement critical in NAFLD pathogenesis. The clock controlled cytoplasmic polyadenylation element binding protein 4 (CPEB4), necessary for adaptation to ER stress triggered by high fat diet, is regulated by the UPR through upstream open reading frames (uORFs) within the 5′UTR and turns on a second wave of UPR translation required for the maintenance of ER and mitochondrial homeostasis (Maillo et al., 2017).

In the presence of ER stress, PERK phosphorylates eukaryotic initiation factor 2α (eIF2α) at Ser51, inducing an overall down regulation of cap-dependent protein translation, whilst mRNAs with uORFs in their 5′-UTR are also translated, such as Atf4 mRNA coding for a transcription factor crucially involved in the stress adaptive response (Maillo et al., 2017). Cpeb4 mRNA encloses several uORFs and is translated in a PERK-dependent manner in face of ER stress (Maillo et al., 2017). In hepatocytes, following ER-stress induced eIF2α phosphorylation, CPEB4 maintains the translation of CPE regulated mRNAs when global protein synthesis is inhibited. The lack of CPEB4-mediated translational control favors high fat diet-induced hepatosteatosis (Maillo et al., 2017). Furthermore, ER stress prompts expression of the hepatokine, fibroblast growth factor (Fgf) 21, triggering PERK and IRE1α/XBP1 signaling pathways, as well as the Atf4 transcription factor (Kim et al., 2013). Atf4 can join two binding sites in the Fgf21 promoter, and is crucial for the expression of *klb* gene, encoding β-Klotho, a divergent structural member of the glycosidase I superfamily (Dong et al., 2015). β-Klotho, expressed in the liver and pancreas and to a lesser extent in adipose tissue, assists Fgf21 in its regulation of insulin-independent glucose transport in adipocytes, body weight and ketogenesis (Bookout et al., 2013). Fgf21 binds a heteromeric cell-surface receptor composed of one of three FGF receptors, (FGFR1c, FGFR2c, or FGFR3c), assembled with β-Klotho in the SCN and the dorsal vagal complex of the hindbrain. Fgf21 restrains the SCN expression of the neuropeptide, vasopressin, thereby impacting the adaptive starvation response through augmented systemic glucocorticoid levels, reduced physical activity, and modified circadian behavior (Bookout et al., 2013). Furthermore, Fgf21 interacts with PPARα in NAFLD pathogenesis. PPARα is triggered in the fasted state, activating ketogenesis and Fgf21 expression, which in adipose tissue maintains glucose uptake and controls lipases essential for fatty acid release, as well as the expression of PPARα target genes directly engaged in fatty acid β-oxidation (Montagner et al., 2016).

### The mitochondrion and the biological clock

Mitochondrial function is circadianly regulated. The Arntl-driven activity of the NAD+-dependent deacetylase, sirtuin 3, regulates 24-h rhythmicity in the acetylation and activity of oxidative enzymes and mitochondrial respiration (Peek et al., 2013). Further circadian regulation is mediated by Per1 and Per2, which control the rate-limiting mitochondrial enzymes involved in lipid and glucose metabolism (Neufeld-Cohen et al., 2016), whilst numerous mitochondrial proteins engaged in metabolic pathways are greatly impacted by clock-driven acetylation (Masri et al., 2013). Mitochondrial respiratory activity shows time-related and Arntl-dependent changes linked to the reversible acetylation of a single subunit of the mitochondrial respiratory chain Complex I driven by cellular NAD+ content fluctuation and clock gene-dependent expression of NAMPT and sirtuins 1/3. Whilst pharmacological inhibition of the mitochondrial oxidative phosphorylation system leads to a marked deregulation of the molecular clockwork (Cela et al., 2016; Scrima et al., 2016). Furthermore, in the mouse liver Arntl organizes mitochondrial biogenesis and dynamics, above all fission and mitophagy, in tune with the rhythmicity of metabolism and bioenergetic fluxes related to the fed and the fasted states. Liver-specific Arntl knockout mice show accumulating oxidative damage and hepatic insulin resistance (Jacobi et al., 2015).

### Gut microbiota modulates the biological clock

NAFLD pathogenesis and progression to NASH are impacted by intestinal host-microbiota interactions. The microbiome shows circadian variation in the spatial/temporal composition and distribution and alterations in these biogeography oscillations, through dysbiosis-induced increased gut endothelial barrier permeability, lead to enhanced systemic bacterial translocation as well as intestinal and hepatic inflammation (Mukherji et al., 2013; Thaiss et al., 2014, 2016; Leone et al., 2015). In turn, these alteration impacts on metabolic homeostasis and the absence of the inflammasome in the intestines disrupts the influence of the microbiome during diet-induced obesity and liver disease (Mukherji et al., 2013; Thaiss et al., 2014, 2016; Leone et al., 2015). The molecular clockwork of intestinal epithelial cells modulates the microbiota-gut barrier interplay. The molecular clockwork also modulates interactions between bacterial products and Toll-like receptors (TLRs), as well as the opposing phases of RORα and Rev-erbα that drive TLR circadian rhythms. Such processes therefore act to translate arrhythmic microbiota signaling into rhythmic JNK and NF-kB signaling activation (Mukherji et al., 2013; Thaiss et al., 2014, 2016; Leone et al., 2015). Interestingly, experiments performed in mouse models fed a methionine-choline-deficient diet to induce liver steatosis show intestinal NLRP6 and NLRP3 inflammasomes, and their product IL-18, to negatively impact the transition from NAFLD to NASH (Henao-Mejia et al., 2012). The loss of these inflammasomes and their products induces changes of the distribution of gut microbiota biogeography as well as the levels of influx of TLR4 and TLR9 agonists into the portal circulation, with the concurrent increase in hepatic tumor-necrosis factor (TNF)-α expression driving NASH progression (Henao-Mejia et al., 2012).

**Figure 3:**
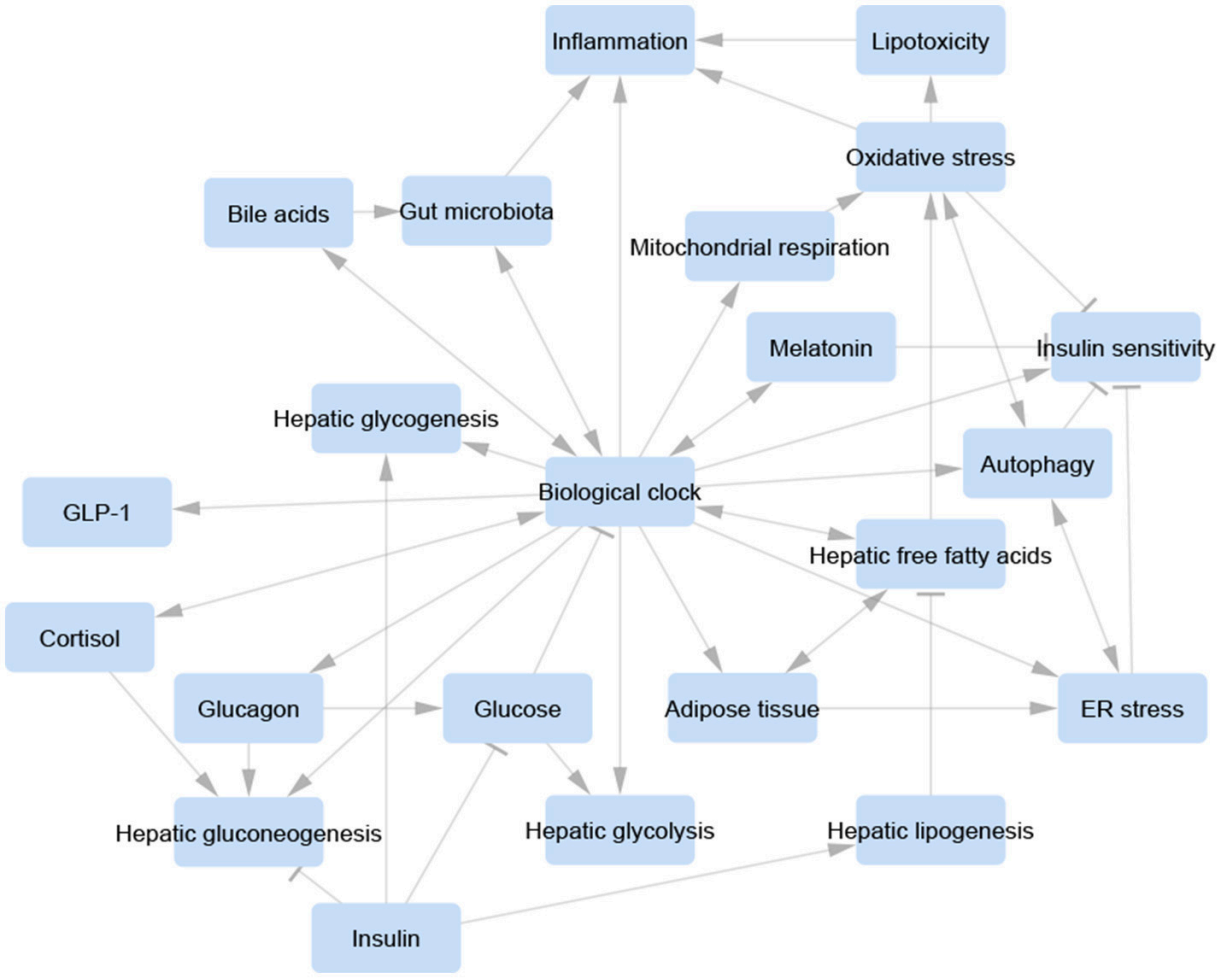
Visualization of a unifying concept of the involvement of the biological clock in the control of the relevant pathways and biological processes involved in non-alcoholic fatty liver disease pathogenesis. Each pathway/process is represented as a blue-rounded rectangle. Arrow-headed lines represent activation, bar-headed lines represent inhibition. Molecular interactions were manually retrieved and curated from the scientific literature. Graphical layout was obtained by Cytoscape 3.4.0 (www.cytoscape.org/).

Appropriate symbiosis between microbiota and intestinal epithelial cells avoids Rev-erbα activation by PPARα and regulates host metabolite oscillations as well as circadian transcriptional and epigenetic landscapes in both the intestine and liver (Mukherji et al., 2013; Thaiss et al., 2014, 2016; Leone et al., 2015). In contrast, microbiota signaling disorder triggers a prediabetic syndrome due to ileal corticosterone excessive secretion resulting from derangement of the biological clock. Indeed, host molecular clockwork dysfunction or jet lag induction provokes anomalous microbiota circadian variations and dysbiosis, prompted by altered feeding rhythmicity (Mukherji et al., 2013; Thaiss et al., 2014, 2016; Leone et al., 2015). Accordingly, a fecal-microbiome-derived metagenomic signature in NAFLD patients was shown to be an important predictor of the classification and severity of fibrotic liver disease (Loomba et al., 2017; Figure 3).

## The molecular clockwork and hepatosteatosis: beyond insulin resistance

NAFLD, without hyperglycemia or insulin resistance, may arise from a variety of different mechanisms, including triglyceride secretion hindrance, subcellular lipid hijacking, decreased lipolysis, increased lipogenesis, gluconeogenesis faults, and fatty acid β-oxidation interference (Sun and Lazar, 2013). Excessive lipogenesis weakens gluconeogenesis, redirecting precursors, and decreasing glycogen storage with resultant hypoglycemia (Sun and Lazar, 2013). In contrast, gluconeogenesis hindrance forces intermediates into lipogenesis and promotes hepatic steatosis. Impediments in lipid β-oxidation lead to triglyceride accumulation, whilst also obstructing gluconeogenesis due to associated shortages in energy and cofactors (Sun and Lazar, 2013).

Hepatic steatosis with systemic insulin hypersensitivity is also associated with FGF21 transactivation by the aryl hydrocarbon receptor (AHR), indicating that the AHR-FGF21 endocrine signaling pathway is a critical environmental transducer that integrates chemical and nutrient signaling in the liver (Lu et al., 2015). The FGF21 promoter contains several putative dioxin response elements (DREs). The AHR-ARNT heterodimer can bind to specific DREs that have overlapping binding sequences for PPARα, as well as for the carbohydrate response element-binding protein (ChREBP), and the cAMP response element-binding protein, hepatocyte specific (CREBH) (Cheng et al., 2014; Girer et al., 2016). The AHR, a member of the basis-helix-loop-helix, PAS domain (bHLH-PAS) family, acts as a ligand-activated transcription factor. The AHR interacts with the biological clock and binds xenobiotics, chiefly planar aromatic hydrocarbons and polyphenols, as well as endobiotics, including endogenous reactive by-products of normal metabolism such as indigoids, heme metabolites, eicosanoids, and tryptophan derivatives (Anderson et al., 2013). The interplay between the AHR/ARNT complex and the circadian network is also important to the increasingly recognized role played by these compounds, also called endocrine-disrupting or metabolism-disrupting chemicals, in NAFLD pathogenesis (Foulds et al., 2017). Such factors act on endocrine and metabolic organs through the AHR-dependent signaling pathway, among other nuclear hormone receptors, with their early-life exposure proposed to prime hepatosteatosis development and progression in adulthood (Foulds et al., 2017).

Experiments performed in AHR liver-specific and inducible transgenic (TG) mice [tetracycline responsive element (TetRE)–constitutively activated human AHR (CA-AHR) TG mice] fed an obesogenic diet showed that AHR activation worsens hepatic steatosis, but inhibits diet-induced obesity and systemic insulin resistance. The metabolic effects of the AHR are mediated through its direct transcriptional target FGF21 (Lu et al., 2015). The metabolic phenotypes shown in CA-AHR TG mice fed chow or a high fat diet could also be related to decreased expression of the circadian genes Clock, Per1, and Per2 with derangement of the biological clock contributing to the dissociation between fatty liver and insulin resistance (Vinciguerra and Mazzoccoli, 2016). The metabolic and protective effects of FGF21 were also shown in liver-specific SIRT1 knockout mice and their wild-type littermates when fasted as well as following SIRT1 activation or fasting mimicking chemical agents (Li et al., 2014). FGF1 also shows circadian expression and positive metabolic effects. FGF1 is regulated by the lipid sensor PPARγ and is crucial for adipose tissue function. Likewise FGF15/19, driven by FXR, may also have a significant role in these processes, including from its impact on bile acid metabolism (Nies et al., 2016).

In normal physiology, lipid and carbohydrate metabolism in the liver are circadianly coordinated with behavioral cycles, *via* the biological clock driving the proportionate rhythmic flow of metabolic intermediates between lipid synthesis and glucose production (Sun and Lazar, 2013). Lipids synthesized in the liver or deriving from other sources are included in lipid droplets before secretion or β-oxidation. Lipotoxicity ensues when lipids overload, thereby overwhelming the subcellular lipid sequestration capacity. In diurnal organisms, intermediary metabolites fueled by carbohydrate catabolism are incorporated into lipids in the fed state coinciding with daytime wakefulness and activity, whereas intermediates are redirected toward glucose production, a process propelled by lipid β-oxidation, in the fasted state associated with night-time sleep and rest (Sun and Lazar, 2013).

The expression of genes enriching lipid metabolic pathways is largely regulated by PPARα, a ligand-activated transcription factor of the steroid hormone receptor superfamily expressed mostly in tissues with high fatty acid catabolism, such as the liver, heart, and muscle. PPARα drives expression of genes encoding enzymes engaged in lipoprotein and lipid metabolism, through fatty acid uptake, binding, and activation [fatty acid transport protein (FATP), fatty acid translocase (FAT/CD36), liver cytosolic fatty acid-binding protein (L-FABP), acyl-CoA synthetase (ACS), carnitine palmitoyltransferase I and II (CPT-1 and CPT-II)], mitochondrial β-oxidation [very long chain acyl-CoA dehydrogenase (VLCAD), long chain acyl-CoA dehydrogenase (LCAD), medium chain acyl-CoA dehydrogenase (MCAD), short chain acyl-CoA dehydrogenase (SCAD)], peroxisomal β-oxidation [acyl-CoA oxidase (ACOX), bifunctional enzyme (HD), 3-ketoacyl thiolase (Thiolase)], microsomal ω-hydroxylation [Cytochrome P450 4A1 and 4A6 (CYP4A1 and CYP4A6)], hydrolysis of plasma triglycerides [lipoprotein lipase (LPL), apolipoprotein C-III (ApoC-III)], fatty acid synthesis [acetyl-CoA carboxylase (ACC), fatty acid synthase (FAS)], liver ketogenesis [HMG-CoA synthase (HMGCS)], fatty acid interconversion [tearoyl-CoA desaturase (SCD)], HDL metabolism [apolipoprotein A-I and A-II (ApoA-I and ApoA-II), ATP-binding cassette transporter 1 (ABCA1)], electron transport chain [uncoupling protein 1, 2, and 3 (UCP1, UCP2, and UCP3)] (Yoon, 2009; Mazzoccoli et al., 2012, 2014).

PPARα can bind endogenous ligands (fatty acids and fatty acid-derived compounds such as modified fatty acids, conjugated fatty acids, oxidized phospholipids, and fatty acid-derived eicosanoids (for example 8-S-hydroxytetraenoic acid and leukotriene) and synthetic ligands (carbaprostacyclin, non-steroidal anti-inflammatory drugs, pirinixic acid, phthalate ester plasticizers, and hypolipidemic drug fibrates, including fenofibrate, gemfibrozil, clofibrate, and ciprofibrate; Yoon, 2009). PPARα expression has a 24-h rhythmicity, driven by Arntl (Canaple et al., 2006), which heterodimerizes with Clock and transactivates PPARα target genes (Inoue et al., 2005). In turn, PPARα acts to regulate Arntl expression, directly binding PPAR response elements (PPRE) located at 1519 and 45 base pairs upstream of the transcription initiation site in the *Arntl* promoter. Accordingly, fenofibrates are capable of prompting *Arntl* expression in Rat-1 fibroblasts *in vitro* and in the mouse liver *in vivo* (Canaple et al., 2006).

Another member of the nuclear receptor ligand-activated transcription factors superfamily, PPARγ, is driven by the biological clock and is a molecular target for thiazolidinediones. PPARγ contributes to the control of adipogenesis, as well as glucose and lipid metabolism, and can bind prostaglandins and eicosanoids. PPARγ positively impacts on Arntl expression by binding its promoter at the same PPRE site, at −1,519 bp, which is involved in the response to PPARα activators (Wang et al., 2008). Experiments performed in Rat-1 fibroblasts *in vitro* and in the mouse liver have also shown that Arntl expression is controlled by the transcriptional repressor, and member of the 3 zinc finger family of Krüppel-like transcription factors (KLF), KLF10 (also known as TGFβ Inducible Early Gene-1, TIEG1). KLF10 senses intracellular glucose levels and joins two juxtaposed GC boxes near the *Arntl* gene transcription initiation site, operating in addition to RORE-dependent repression by Rev-erbα (Hirota et al., 2002, 2010). Arntl drives the 24-h periodicity of KLF10 expression, which in the mouse liver regulates the circadian variation of genes involved in lipogenesis, gluconeogenesis, and glycolysis (Guillaumond et al., 2010) The reciprocal control between PPARα/Arntl, PPARγ/Arntl, and KLF10/Arntl allows fine tuning of metabolic fluxes and determines “acceleration and braking,” thereby redirecting intermediary metabolites through the different metabolic pathways in line with the biochemical and behavioral rhythms related to feeding/fasting and waking/resting cycles, driven by the circadian timing system. Nutrient excess or feeding mistiming may overwhelm the liver handling capacity and cause imbalance between lipid and glucose metabolism, with ensuing hepatosteatosis.

## Therapeutic strategies for NAFLD and NASH impacting the biological clock

The increasing worldwide epidemic of NAFLD and NASH, the association with multiorgan pathology and the lack of valuable pharmacological strategies, motivate research into ground-breaking pathophysiological mechanisms, leading to innovative therapeutic approaches for this pathology. The clinical and histological phenotypes of NAFLD and NASH are comparable worldwide, while the epidemiology and demographic characteristics vary in different countries around the world and proper categorization of NAFLD in line with comprehensive classifiers could afford more effective therapeutic and preventive strategies. Insulin resistance is associated with being overweight, and is a central pathogenetic mechanism in the majority of NAFLD cases in the Western world, whereas the disease is to a lesser extent associated with obesity and insulin resistance in the Eastern world (Loomba and Sanyal, 2013).

### Dietary intervention

Lifestyle modification, especially dietary habits, changes calorie restriction, and physical exercise are recommendation to help improve hepatic steatosis and is the standard of care of NAFLD as well as the first step for the treatment of NAFLD metabolic comorbidities. As a minimum 3–5% of weight loss, achieved by hypocaloric diet alone or in combination with physical activity and behavioral modification, commonly diminishes hepatic steatosis, whilst at least 10% of weight loss may be needed to ameliorate hepatic necroinflammation (Lazo et al., 2010; Fan and Cao, 2013).

As well as dietary patterns, referring to nutrient quality and/or quantity in diets, time-related feeding patterns, including frequency and time-of-day during which meals are habitually eaten (feeding), or in contrast, not consumed (fasting), also play a role in determining metabolic disorder onset/progression and could be addressed for therapeutic purposes (Mattson et al., 2014). This issue was mentioned across centuries by numerous historical figures and health professionals and extensively addressed in his book titled “The No Breakfast Plan (and The Fasting-Cure)” published back in 1900 by Edward Hooker Dewey, an American physician considered a forerunner in proposing the curative effects of fasting. Interestingly, positive and pleiotropic metabolic effects have been corroborated by several scientific investigations for intermittent fasting, periodic fasting, and fasting mimicking diet as well as time restricted feeding (Longo and Panda, 2016; Table 2). Feeding behavior and in particular time-of-day at which dietary fat is consumed significantly impacts on mouse weight gain, adiposity, glucose intolerance, hyperinsulinemia, hypertriglyceridemia, and hyperleptinemia, independent of daily total or fat-derived calories (Bray et al., 2010). Experiments performed in mice fed a high-fat high-sucrose diet show that short-term feeding mistiming (food consumption during daytime in nocturnal animals) desynchronizes peripheral clocks and provokes metabolic derangement with hepatic fat accumulation, hypothermia, and reduced physical activity (Yasumoto et al., 2016). Conversely, time-restricted feeding of a high-fat diet for 8 h during nighttime induces mice to eat equivalent calories, in respect to controls with *ad libitum* access to food, but the former show enhanced CREB, mTOR, and AMPK pathway signaling, increased oscillations of core and clock controlled genes, and do not develop obesity, hyperinsulinemia, hepatic steatosis, and inflammation, whilst also showing superior motor coordination (Hatori et al., 2012; Chaix et al., 2014).

Animal experiments performed in lean C57BL/6 and BALB/c mice kept at room temperature or at a mouse thermoneutral zone (30°C) as well as in obese leptin-deficient mice, show that calorie restriction triggers functional beiging of subcutaneous and visceral white adipose depots and improves metabolism with negative energy balance, whilst also amplifying eosinophil infiltration, type 2 cytokine signaling, and M2 macrophage polarization in fat (Fabbiano et al., 2016). Mice fed a regular-chow diet and subjected to cycles of every-other-day fasting showed that intermittent fasting maintains white adipose tissue beiging by means of gut microbiota modulation, with striking augmentation of Firmicutes loads and the Firmicutes to Bacteroidetes ratio (Li et al., 2017). This leads to a lessening of visceral white adipose tissue mass, conspicuous beiging of subcutaneous white adipose tissue depot, augmentation of multilobular adipocytes (a beige adipocytes hallmark) and outstanding Ucp1 up-regulation in subcutaneous white adipose tissue, all leading to increased metabolism and lower weight gain (Li et al., 2017). Furthermore, in obese mice fed a high-fat diet, every-other-day fasting significantly decreased obesity, insulin resistance and hepatic steatosis, suggesting intermittent fasting as a feasible drug-free intervention for NAFLD and dysmetabolism in the context of obesity (Li et al., 2017). Interestingly, mice exposed to caloric restriction strengthen their food intake behavior and carry out spontaneous time-restricted feeding with enhanced wheel-running activity in their rest period. Such results hint at links among feeding, metabolism, and behavior (Acosta-Rodríguez et al., 2017). Furthermore, the biological clock in mouse liver and gut is impacted by ketogenic diet, inducing overproduction of acetyl-CoA and increased ketone bodies synthesis, through ketogenic pathway activation (McHill et al., 2017). High-throughput transcriptome analysis of liver and gut specimens harvested from normal chow, or ketogenic diet fed 8-week-old C57BL/6 mice, showed that clock-controlled genes and Arntl chromatin recruitment were significantly increased only in the liver in the latter group (McHill et al., 2017). PPARα nuclear accumulation, as well as target gene expression, was increased in both tissues although with a diverse circadian phase. Finally, a ketogenic diet prompts a strong 24-h fluctuation of serum and intestinal β-hydroxyl-butyrate levels, triggering tissue-specific cyclic HDAC activity and histone acetylation (McHill et al., 2017).

In humans, feeding time is a recently pinpointed risk factor for dysmetabolism, impacting body composition notwithstanding more long-established risk factors (for example amount or content of food intake and activity level). The timing of food intake relative to melatonin onset is significantly associated with the percentage of body fat and body mass index (Tognini et al., 2017). Late-night dinner eating coinciding with a rising endogenous melatonin concentrations leads to impairment of glucose tolerance, especially in homozygous carriers, vs. non-carriers, of the MTNR1B risk allele (Lopez-Minguez et al., 2017). Besides, melatonin administration (5 mg) at different time-of-day (morning vs. evening) negatively impacts on glucose tolerance evaluated by an oral glucose tolerance test both in the morning and evening in healthy subjects (Rubio-Sastre et al., 2014), but is influenced by the genotype and is evident only in the morning in carriers of the MTNR1B genetic variant rs10830963 (Garaulet et al., 2015). Taken together, these studies hint that eating at a later circadian time is associated with increased body fat and impaired glucose tolerance, suggesting a critical role played by human chronotype and genotype in the interplay among time-related feeding patterns, endogenous circadian time, hormone secretion, content of food intake and body composition (Rubio-Sastre et al., 2014; Garaulet et al., 2015; Lopez-Minguez et al., 2017; Tognini et al., 2017).

### Pharmaceutical intervention

Evidence-based clinical practice guidelines recommend pharmaceutical compounds with a primary metabolic target in NAFLD patients with advanced liver disease or at high risk of progression to cirrhosis. Some of these molecules impact the biological clock and include: PPAR agonists (clofibrate, fenofibrate, bezafibrate, gemfibrozil, pemafibrate, LY518674, elafibranor, saroglitazar, pioglitazone,rosaglitazone), FXR agonists (obeticholic acid, GS-9674), bile acid sequestrants (sevelamer), GLP-1 receptor agonists (liraglutide), FGF-21 analogs (BMS-986036), FGF-19 analogs (NGM-282), SIRT1 activating agents (resveratrol, SRT1720), gut microbiota modulators such as antibiotics (solithromycin) and fecal microbial transplant (Chalasani et al., 2012; Gross et al., 2017; Rotman and Sanyal, 2017). Other molecules targeting specific clock components (He et al., 2016; Chen et al., 2018) need careful evaluation of their utility in the prevention or treatment of NAFLD, through clinical trials assessing the long-term safety and efficacy of these therapies.

## Conclusions

NAFLD, the most common hepatic disease in the Western world, is caused by deranged hepatic free fatty acid trafficking and metabolism and may proceed to NASH, leading to cirrhosis, portal hypertension and eventually liver cancer. The pathophysiology of NAFLD includes alterations in nutrient fluxes, bile acids turnover, nuclear receptors ligand binding, gastro-intestinal insulinotropic hormone secretion, microbiota-gut barrier interaction, autophagy, and the immune/inflammatory cascade. These processes are managed by the biological clock and disruption of their circadian synchronization and phase resonance with behavioral alternation of feeding/waking and fasting/resting provokes immune-metabolic dysregulation (Reinke and Asher, 2016; Tahara and Shibata, 2016). Chronic metabolic stress, related to ongoing nutrient surplus or mistimed feeding, overwhelms the fine-tuning of biochemical mechanisms, so that lipid influx engulfs the adipose tissue and causes the amassing of damaging lipid species in the liver and muscle. Lipotoxicity interferes with metabolic and immune/inflammatory signaling pathways and causes a pathological process defined as “metaflammation” that underlies NAFLD to NASH progression. Therapeutic approaches including time-related dietary patterns (chrononutrition) and life habits changes, as well as addressing the molecular clockwork and the circadian timing system are under evaluation. Further investigation of these factors may provide innovative strategies for the treatment of metabolic disorders and hepatosteatosis (Table 3). A more comprehensive knowledge of the molecular signaling pathways involved in NAFLD pathogenesis, as well as in NAFLD to NASH progression, could open the way to ground-breaking science-based strategies for prevention and therapy, such as pharmaceuticals directly targeting the biological clock or acting through nuclear receptors, bile acid signaling or gut microbiota modulation.

**Table 3:** NAFLD and the biological clock: new perspectives and unanswered questions.

What is the impact of light-at-night on NAFLD epidemiology in the general population and in shift-workers? Socially driven circadian dysregulation could be evaluated in relation to global prevalence, incidence, progression, and outcome of hepatic steatosis.
What are the effects of ecological light pollution? Night-time exposure to blue light from electronic screens and lighting could have health consequences. The amount of night-time use of such devices after dusk could have long-lasting metabolic consequences.
How could the metabolic consequences of socially driven circadian dysregulation be lessened? Proper planning of school and work schedules as well as forward vs. backward shift rotation could have significant effects on the internal timing organization and metabolism, thereby contributing to the maintenance of health, including the lowering of hepatosteatosis.
Employees who work nighttime or rotating shifts and night-eaters have altered time-related dietary patterns: re-entrainment of the biological rhythms by more appropriately timed feeding schedules as well as intermittent and periodic fasting or fasting-mimicking diets, could be a valuable approach to prevent or ameliorate metabolic derangements.
Could time-restricted feeding or intermittent fasting help in managing the adverse metabolic effects induced by exposure to light-at-night or socially driven circadian dysregulation?
How does chronodisruption recovery improve the metabolic derangements underlying NAFLD pathogenesis? Light therapy, chrononutrition, or late evening melatonin administration could be helpful intervention strategies.
How could shift workers cope with exposure to light-at-night in their work environment? Intervention strategies could include supplying workers with goggles and luminaire to avoid exposure to light wavelengths below 530 nm, to prevent suppression of nocturnal melatonin secretion and to restrain chronodisruption.
How do sleep disorders impact metabolism and what is the role of the biological clock? The interplay among the sleep/wake homeostat and the circadian timing system remains largely unexplored. An increased risk of NAFLD was shown in a middle-aged population with short sleep duration and poor sleep quality.

## Author contributions

GM, SD, and TM: manuscript design, writing, revision; TM: bioinformatics analysis and figures drawing; GM, SD, and TM: approval of the final version of the manuscript.

### Conflict of interest statement

The authors declare that the research was conducted in the absence of any commercial or financial relationships that could be construed as a potential conflict of interest.
